# Beyond the ruler: Measuring what truly matters in endoscopic ultrasound-guided pancreatic cancer genomics

**DOI:** 10.1055/a-2677-0505

**Published:** 2025-08-14

**Authors:** Jinyu Wu, Wen Wang, Kuncheng Yang

**Affiliations:** 1 Department of Gastroenterology, The First People's Hospital of Shuangliu District (West China Airport Hospital of Sichuan University), Chengdu, China; 2 The Second Affiliated Hospital of Chengdu Medical College (Nuclear Industry 416 Hospital), Chengdu, China; 3 Department of Dermatology, The People's Hospital of Rongchang District, Chongqing, China

We read with great interest the article by Sato et al. entitled “Benefits of Macroscopic On-Site Evaluation in Endoscopic Ultrasound-Guided Tissue Acquisition for Comprehensive Genomic Profiling” 1 . The study highlights the importance of macroscopic visible core (MVC) length in determining the success of FoundationOne CDx (F1CDx) analysis, proposing a threshold of 42 mm for optimal sample adequacy. Although this research provides valuable insights, we would like to raise two critical points for further discussion.

First, the study’s primary outcome relies on the categorization by Foundation Medicine Inc. (Passed, Qualified, or Failed), yet the specific criteria for these classifications remain unclear. Although a 42-mm MVC length correlates with technical success, the clinical implications of qualified samples – particularly their impact on mutation detection rates, subsequent treatment adjustments, and patient survival outcomes – are not addressed. For precision medicine to be truly actionable, it is essential to bridge this gap by tracking the entire pathway from sample acquisition to therapeutic response. Future studies should focus on establishing thresholds based on clinically actionable mutation detection rates rather than technical adequacy alone.

Second, the study included 17% of patients who had undergone prior chemotherapy but did not account for the potential biological impact of treatment on tumor heterogeneity. Chemotherapy may eliminate chemosensitive clones while enriching resistant subclones 2 , leading to sampling bias in post-treatment biopsies. This could result in necrotic or fibrotic tissue, altering the required sample size compared with untreated tumors. We recommend a subgroup analysis of chemotherapy-naïve and post-chemotherapy patients to derive distinct MVC length thresholds, ensuring more accurate genomic profiling in both settings.

In conclusion, this study represents a significant step toward optimizing endoscopic ultrasound-guided tissue acquisition for comprehensive genomic profiling. However, to fully realize the promise of precision medicine, future research must align technical parameters with clinical outcomes, ensuring that genomic data translates into tangible patient benefits.

**Publication note**

Letters to the editor do not necessarily represent the opinion of the editor or publisher. The editor and publisher reserve the right to not publish letters to the editor, or to publish them abbreviated or in extracts.
